# Periprosthetic Infections of the Shoulder Joint: Characteristics and 5-Year Outcome of a Single-Center Series of 19 Cases

**DOI:** 10.3390/antibiotics10091125

**Published:** 2021-09-18

**Authors:** Mohamad Bdeir, Franz-Joseph Dally, Elio Assaf, Sascha Gravius, Elisabeth Mohs, Svetlana Hetjens, Ali Darwich

**Affiliations:** 1 Department of Orthopaedic and Trauma Surgery, University Medical Centre Mannheim, Medical Faculty Mannheim, University of Heidelberg, Theodor-Kutzer-Ufer 1-3, 68167 Mannheim, Germany; mohamad.bdeir@umm.de (M.B.); franz.dally@umm.de (F.-J.D.); elio.assaf@umm.de (E.A.); sascha.gravius@umm.de (S.G.); elisabeth.mohs@umm.de (E.M.); 2 Institute of Medical Statistics and Biomathematics, University Medical Centre Mannheim, Medical Faculty Mannheim, University of Heidelberg, Theodor-Kutzer-Ufer 1-3, 68167 Mannheim, Germany; svetlana.hetjens@medma.uni-heidelberg.de

**Keywords:** periprosthetic joint infection, PJI, shoulder, PSI, characteristics, outcome, case series

## Abstract

Periprosthetic shoulder infection (PSI) remains a devastating complication after total shoulder arthroplasty (TSA). Furthermore, there is a paucity in the literature regarding its diagnostic and therapeutic management, especially the absence of therapy concepts devised exclusively for PSI. The aim of the presenting study is to examine the characteristics and outcome of patients with PSI who were treated according to well-established algorithms developed originally for periprosthetic joint infection (PJI) of the hip and knee and determine if these algorithms can be applied to PSI. This single-center case series included all patients with a PSI presenting between 2010 and 2020. Recorded parameters included age, sex, affected side, BMI, ASA score, Charlson comorbidity index, preoperative anticoagulation, indication for TSA (fracture, osteoarthritis or cuff-arthropathy), and type of infection (acute or chronic PSI). The outcome was divided into treatment failure or infect resolution. Staphylococcus epidermidis and aureus were the commonest infecting pathogens. Acute PSI was mainly treated with debridement, irrigation, and retention of the prosthesis (DAIR) and chronic cases with two/multiple-stage exchange. The treatment failure rate was 10.5%. C-reactive protein was preoperatively elevated in 68.4% of cases. The mean number of operative revisions was 3.6 ± 2.6, and the mean total duration of antibiotic treatment was 72.4 ± 41.4 days. The most administered antibiotic was a combination of clindamycin and fluoroquinolone. In summary, the data of the current study suggest that therapeutical algorithms and recommendations developed for the treatment of PJI of the hip and knee are also applicable to PSI.

## 1. Introduction

Total shoulder joint arthroplasty (TSA) experienced in the last years’ considerable advancements and is nowadays a well-established treatment option for various diagnoses such as proximal humerus fracture, osteoarthritis, and cuff tear arthropathy in the elderly [1,2,3]. The increasing number of performed TSA is accompanied by growing complication rates such as periprosthetic shoulder infection (PSI), where incidence estimates of 0.7 to 6% are reported [4,5,6,7]. PSI is considered one of the most devastating complications of TSA and a common cause of surgical revision and persistent shoulder pain [8]. Not only it constitutes a great burden to the health care system, but it is also associated with unsatisfactory functional outcomes and impairment [4].

The most commonly identified microorganisms in PSI are Cutibacterium acnes and coagulase-negative Staphylococcus [8,9,10,11], in contrast to the periprosthetic joint infection (PJI) of the hip and knee in which mostly Staphylococcus aureus is detected [12]. Cases with low-virulence pathogens pose a challenge in addition to delayed diagnosis with resulting delayed therapy [13]. Although, on the one hand, the spectrum of infecting microorganisms in periprosthetic joint infection (PJI) vary between the shoulder and the hip/knee and on the other hand, significant anatomical and biomechanical differences are present, management of PSI and the different modalities of surgical therapy are often based on guidelines for PJI of the hip or knee. These include debridement, irrigation, and retention of the prosthesis (DAIR), one-, two- or multiple-stage exchange and resection arthroplasty [14,15,16,17,18].

Several studies investigated the development of PSI [6] and documented an association with numerous risk factors such as previous shoulder surgery [19,20], higher age [21,22], male sex [20,22], increasing body mass index (BMI) [20,23], diabetes mellitus [19,20,23], radiation therapy [19], use of steroids [23,24] and malignancy [23,24]. However, factors affecting treatment failure in PSI are not intensively investigated [6]. In fact, only the isolation of Cutibacterium acnes [6,25], smoking [26], and increasing BMI [27] were observed to have a negative effect on the complication rates and patient outcomes. Concerning the effect of PSI on the patient end outcomes, there is a paucity of data in the literature [28].

The aim of the presenting study is to examine the characteristics, and outcome of patients with PSI who were treated according to well-established algorithms developed originally for periprosthetic joint infection (PJI) of the hip and knee and determine if these algorithms can be applied to PSI.

## 2. Results

Between 2010 and 2020, a total of 19 patients presented with a PSI were included in this retrospective single-center case series. Data regarding demographic characteristics, treatment strategies, and outcomes for included patients are summarized in Table 1.

The series involved 11 females and 8 males with a mean age of 66.1 ± 11 years (range 48–93 years), a mean BMI of 27.8 ± 5.8 kg/m^2^ (20.2–39.1 kg/m^2^), a mean age-adjusted CCI of 4.2 ± 2.3 (1–9) and a median ASA score of 2 ± 0.5 (2–3). A total of eight patients (42.1%) presented to the hospital under anticoagulation. The mean preoperative serum CRP level at admission was 74.7 ± 99 mg/L (5–331 mg/L). TSA was performed in 10 patients (52.6%) for fracture of the proximal humerus and osteoarthritis or cuff-arthropathy for the rest. A total of 12 (63.2%) patients presented with an acute PSI. In three patients, a PSI-related surgical treatment was performed in an external hospital prior to the admission to our hospital. The same three patients were under antibiotic treatment at the time of admission. In six patients (31.6%), a preoperative joint aspiration was performed. None of the patients was under antibiotic treatment at the time of aspiration. In four of the six patients, the detected pathogen matched that detected intraoperatively (sensitivity 66.7%). In the remaining two cases, no pathogen was detected in the aspiration, though a causing microorganism was detected in the intraoperative tissue samples.

Staphylococcus species were responsible for 12 of the 19 reported PSI cases (63.2%). Staphylococcus epidermidis was isolated in six cases (31.6%): in four cases (21.1%) as a monomicrobial infection and in two cases (10.5%) along with Enterococcus faecalis and Proteus mirabilis, respectively, as a polymicrobial infection. A total of five of the six cases with Staphylococcus epidermidis involved methicillin-resistant strains. Staphylococcus aureus was also detected in six cases (31.6%) (5 methicillin-susceptible and one methicillin-resistant). In four cases (21.1%), a culture-negative PSI was diagnosed based on clinical and histological findings. Citrobacter freundii, along with Escherichia coli, caused one polymicrobial infection, and Cutibacterium acnes and Escherichia coli caused one monomicrobial infection each.

In 8 patients (42.1%), the prosthesis was retained (DAIR), and in 11 patients (57.9%), a two/multiple-stage exchange was performed. In 8 (72.7%) of these 11 patients, the implant was loose. After implant removal, reimplantation was successful in seven cases (63.6%). Two patients opted for a permanent resection arthroplasty and refused the reimplantation due to advanced age (93 years old) in the first case and multimorbidity (ASA score 3, age-adjusted CCI 5) with a satisfactory range of motion (Abduction of 110°) in the second case. In the two remaining cases, a treatment failure was observed with persistent infection and consequent chronic antibiotic suppression.

The total mean number of operative revisions was 3.6 ± 2.6 (1–12), and the mean total duration of antibiotic treatment was 72.4 ± 41.4 days (28–214 days). A total of 68% of included PSI (13/19) were treated with clindamycin that was initially empirically started to treat Cutibacterium acnes, which, according to literature, was considered as the most common infecting agent in PSI. The combination was mainly with fluoroquinolone.

At a mean total follow-up of 57.6 ± 36.4 months (18–138 months), in 17 patients (89.5%), an infection resolution was observed, and in 2 patients (10.5%), a treatment failure (persistent infection with consequent chronic antibiotic suppression). One PSI-unrelated death has been recorded. Regarding shoulder function, a mean abduction of 61.8° ± 20.5° was reported.

## 3. Discussion

PSI remains a devastating complication after TSA, with a prevalence ranging from 1% to 19% after primary TSA and up to 15% after revision surgeries [29,30,31]. However, there is a paucity in the literature regarding its diagnostic and therapeutic management, and the main treatment protocols are often based on the more extensively investigated guidelines for PJI of the hip or knee joint [32,33,34].

In the absence of therapy concepts devised exclusively for PSI at the time, the study was in progress, the patients with PSI included in this study were treated according to the well-established algorithms developed originally for PJI of the hip and knee. The aim of the presenting study was to examine the characteristics and 5-year outcome of these patients and determine if these opted algorithms can be applied to the shoulder joint.

In the present study, the most commonly detected causative pathogens were Staphylococcus epidermidis and Staphylococcus aureus, each in 31.6% of cases. Cutibacterium acnes was detected in one case (5.3%). This goes in line with data in the literature, where low-virulence microorganisms such as coagulase-negative Staphylococci are reported as the commonest infecting agents followed by Staphylococcus aureus [23,35]. However, the rate of infections with Cutibacterium acnes is lower than those reported in the literature, where infection rates of up to 32.5% are documented [36]. This may be due to the female predominance (58%) observed in the current study since the colonization with Cutibacterium acnes is known to be greater in men than in women [37].

In 5 cases where an implant loosening was intraoperatively observed, the PSI was classified as acute. The accuracy of this classification was questionable since implant loosening is a classical feature of chronic or delayed and especially low-grade infections caused typically by low-virulence pathogens [38]. Further analysis of these 5 cases showed that all of them were classified as acute based on the subjective complaints of the patients and their statements regarding the time of appearance of symptoms. None of the cases were PSI appearing within 4 weeks after primary implantation. This implies that inaccuracies in the statements provided by the patients may have had an effect on the classification of the infections. On the other hand, the classification of periprosthetic infections in acute or chronic and defining the specific time limit cut-off has been controversially discussed, and the issue has been raised in the 2018 International Consensus Meeting on Musculoskeletal Infection (MSKI) [39] that concluded that a periprosthetic infection is a continuum and the strict distinction criteria between acute and chronic bone and implant-related infection remain unclear.

In total, in 2 of the 19 cases (10.5%), a treatment failure was reported. The first case was a polymicrobial PSI caused by methicillin-resistant Staphylococcus epidermidis (MRSE), Enterococcus faecalis, and Proteus mirabilis. The poorer outcome of polymicrobial PJI has been shown in the publication of Tan et al. [40], where failure rates of 50.5% were observed, in comparison to 31.5% in monomicrobial PJI and 30.2% in culture-negative PJI. The second case involved an elderly multimorbid patient with an age-adjusted CCI of 5 and a BMI of 30.4 kg/m^2^. Further analysis showed diabetes mellitus as well as malignant disease (Stomach cancer with surgical resection and radiation therapy) in the medical history and positive smoking status. In the publication of Hatta et al. [26] and Wagner et al. [27], the negative effect of smoking and increased BMI (>30 kg/m^2^) on the outcome after TSA was shown.

Regarding diagnostic parameters, CRP is one of the most commonly used biomarkers for a systemic response to inflammation [41]. It is cost-effective, rapid, and implemented in most of the diagnostic algorithms and recommendations for PJI diagnosis [42,43,44]. In the present study, preoperative CRP was elevated in 13/19 cases (68.4%). This correlates with the values reported in the systemic review of Mercurio et al. [36], where CRP was elevated in 70% of the included PSI cases of the 21 analyzed studies.

The surgical treatment in all but two PSI secondary to low-virulence microorganisms (Staphylococcus epidermidis and Cutibacterium acnes) and all but two PSI secondary to Staphylococcus aureus was two/multiple-stage exchange. The eight cases treated with DAIR involved six cases of acute PSI and two cases of chronic PSI.

A mean active abduction of 67.7° ± 20.7° was recorded after multiple-stage protocol versus 53.75° ± 18.5° after DAIR. These results are consistent with those reported by Sperling et al. [23], where the two-stage procedure was found to be the treatment regimen with the best functional outcome. The clinical assessment of the patients in the study of Sperling et al. [23] showed a mean active abduction of 100°; considerably higher than the patients included in the study of Ince et al. [24], where a mean active abduction of 51.6° after the one-stage exchange was reported. However, the abduction values of 53.75° ± 18.5° after DAIR reported in the present study lie lower than those reported by Mercurio et al. [36] and Lemmens et al. [18], where values of 100° and 86°, respectively, were reported. Further analysis of the cases managed with DAIR in the presenting study revealed that these were older (67.4 ± 9.6 years versus 65.2 ± 12.2 years) and with more comorbidities (CCI 4.5 ± 2.7 versus 4 ± 2.1), when compared with patients managed with two/multiple-stage exchange. These two factors may have played a role regarding the poorer functional outcome in the DAIR subgroup. Because of the small number of patients, the results did not reach statistical significance (p 0.3417 and p 0.6158, respectively).

Two cases of chronic PSI were treated contrary to the algorithm with DAIR. Both cases were culture-negative PSI. Otherwise, all other patients that underwent DAIR presented with an acute PSI. The total duration of the antimicrobial treatment in this group was also shorter than that in the two/multiple-stage group with 50.1 ± 13 days (28–70 days) versus 88.6 ± 47.7 days (43–214 days), and the number of revisions was also lower (2.5 ± 1.6 versus 4.4 ± 2.9 revisions). The results correlate with those published by Lemmens et al. [18], where a median antibiotic treatment duration of 6 weeks after DAIR was reported. However, Lemmens et al. [18] did not report a difference in the antibiotic duration between patients after DAIR and two-stage exchange.

The main infecting pathogens detected in the present study, namely Staphylococcus epidermidis and aureus, did not vary from the spectrum of microorganisms commonly found in PJI of the hip and knee [34,45].

A difference to be noted was the varying sensitivity of CRP in diagnosing the periprosthetic infection. In the current study, the sensitivity of CRP was 68.4%, considerably lower than the values reported by van den Kieboom et al. [46], where a sensitivity of 94% in the diagnosis of knee and hip PJI was observed.

To summarize, the data of the current study suggests that the therapeutical algorithms and recommendations developed for the treatment of PJI of the hip and knee are also applicable to the shoulder joint, and published algorithms for the management of PSI do not offer clear guidance regarding indication or provide a superior outcome. According to the latest recommendations of the ICM on orthopedic infections concerning PSI, DAIR was less successful in the treatment of chronic PSI, and there was insufficient high-quality evidence to support or to discourage the use of this approach in the treatment of acute PSI [47]. In addition, there was insufficient data to support the exchange of modular parts during DAIR, especially in acute PSI cases [47]. Nonetheless, in the management of PSI in patients of the current study, the classical recommendations of hip and knee PJI management [48] were applied, and DAIR was mainly performed in acute cases, and mobile modular parts were routinely exchanged. This led to a success rate of 100%, a rate even higher than the reported success rates in the literature in hip and knee PJI, where infection eradication rates of 75.40% and 52.6%, respectively, were reported [49].

Regarding one or two-stage exchange strategies, the indication and guidance algorithm in chronic PSI cases in the ICM was unclear and lacking evidence [47]. In the management of chronic PSI in the current study, the standard recommendations of hip and knee PJI management [48] advising two-stage exchange were applied in five cases. A success rate of 80% was observed, slightly lower than the rate of treatment success in hip and knee PJI of 85.2% documented in the literature [50].

The surgical treatment option of each of the cases reported in the current study was chosen, relying both on the internal algorithms of the hospital and on the individual patient profile. The antimicrobial treatment option was also chosen based on the microbiological findings of each case, and the management of the infection was jointly directed by orthopedic surgeons and infectiologists from the institute of medical microbiology and hygiene in a multidisciplinary context. The patients were examined regularly in close follow-up examinations and for a relatively long period of time in order to detect any sign of infection recurrence as soon as possible. All these factors played an important role in the optimization of PSI management and were responsible for the satisfactory results observed in this study.

One of the limitations of this study is the low level of evidence due to its retrospective and descriptive design. Another limitation is the rather small total sample size, even though the study is a single-center study with a prolonged follow-up that reports on a cohort larger than similar prior studies. A third limitation of this study is the choice of treatment regimen in the analyzed cases. These cases were included over a period of 10 years. Due to this long duration of inclusion, the therapy regimes were obviously subject to further development and improvement based on the evolving and available literature, which may have had a small effect on the course of the infection and created a heterogenicity in the management protocols.

## 4. Materials and Methods

### 4.1. Patient Collective

This case series included all patients with a PSI presenting between 2010 and 2020 to the Orthopedic and Trauma Surgery Centre of the University Medical Centre Mannheim. There were no exclusion criteria. The study has been reported in line with the PROCESS Guideline [51].

### 4.2. Definitions and Parameters

PSI was diagnosed according to the updated and modified criteria of the Shoulder Group of the International Consensus Meeting (ICM) on Orthopedic Infections [47].

Baseline characteristics included age, sex, affected side, and body mass index (BMI). Preoperative patient status was evaluated using the ASA (American Society of Anesthesiologists) score [52] and the age-adjusted form of the Charlson comorbidity index [53]. Further recorded parameters involved preoperative anticoagulation, indication for TSA (fracture, osteoarthritis or cuff-arthropathy), type of infection (acute or chronic PSI), and treatment before admission (antimicrobial or surgical) as well as a preoperative serum level of C-reactive protein (CRP) at admission. In line with the guidelines of the Infectious Diseases Society of America [54], the interval between primary TSA and PSI was divided into acute infections (within 4 weeks after TSA or with symptoms of less than 3 weeks) and chronic infections (occurring after these time limits). It was also documented if a preoperative joint aspiration was performed. The infecting microorganism was recorded as well as the type of surgical and antimicrobial treatment, the total duration of antimicrobials administration (oral and intravenous) in days, the number of revisions performed and the presence of an implant loosening.

The shoulder function was evaluated using the AO neutral-0-method to quantify abduction [55]. Outcomes were categorized based on a modified version of the system proposed by Laffer et al. [56] into:

- Infect resolution: no clinical signs of infection and CRP < 10 mg/L after a minimum follow-up of 18 months;
- Treatment failure: persistent infection or re-infection through the same or a different microorganism with or without the need of a surgical revision, chronic antibiotic suppression, or death due to PSI-related sepsis.

### 4.3. Microbiological and Histological Methods

A preoperative joint aspiration was routinely performed, except in emergency cases or in cases where the infecting pathogen was already identified in a previous surgery. The joint aspirate was first sent to the laboratory to determine cell count as well for cytological differentiation and second for culturing in sterilely inoculated blood culture vials [57]. The ventral or dorsal approach was used to perform the joint aspiration.

Intraoperatively, a minimum of four specimens was collected for microbiological culturing and four specimens for histopathological analysis. Every pair of tissue specimens were obtained from the same anatomical site to match microbiological and histological results. Cultures were considered negative if there was no growth of microorganisms within 10 days [58]. The classification of Morawietz et al. [59] was used to define proof of a PJI in the histopathological examination of the intraoperative samples. After all specimens were collected, a specific or empiric antibiotic treatment was administered, according to whether the identity of the causative microorganism is known or not.

### 4.4. Therapy Regimens

All orthopedic device-related infections in our university hospital are jointly managed by orthopedic surgeons and physicians from the institute of medical microbiology and hygiene. This multidisciplinary treatment concept involves regular rounds to set and control all diagnostic and treatment aspects in order to provide the best treatment of PJI.

The surgical therapy was chosen according to a well-defined internal algorithm and included: debridement, irrigation, and retention of the prosthesis (DAIR), two- or multiple-stage exchange, and resection arthroplasty [34,60,61]. As mentioned earlier, in the absence of therapy concepts devised exclusively for PSI in the study period, the surgical treatment algorithms were based on the extensively investigated concepts developed originally for PJI of the hip and knee [34].

Postoperatively, antibiotics were administered for a period of 6 weeks: intravenously for the first 2 weeks then orally for further 4 weeks under close clinical and laboratory monitoring [62].

Reimplantation was performed only when the local findings (healed surgical wound with no swelling, erythema, tenderness, or discharge from incision site) and the laboratory results (C-reactive protein (CRP) < 10 mg/L) were satisfactory and showed no signs of persistent infection following a 2-week antibiotic-free interval [62].

After reimplantation, biofilm-active antibiotics were administered for a period of 2 weeks intravenously and further 4 weeks orally [63].

## 5. Conclusions

The data of the current study suggests that the therapeutical algorithms and recommendations developed for the treatment of PJI of the hip and knee are also applicable to the shoulder joint provide satisfactory outcomes. The close collaboration between orthopedic surgeons and infectiologists to set and control all diagnostic and treatment aspects is the key element for the successful management of PSI.

## Figures and Tables

**Table 1:** Data regarding demographic characteristics, treatment strategies, and outcomes for patients with periprosthetic infection of the shoulder.

Patient Number	Sex	Age (Years)	Side	BMI (kg/m^2^)	ASA Score	CCI Age-Adjusted	CRP at Admission (mg/L)	Preoperative Anticoagulation	Indication for Prosthesis	Type of Infection	Surgical Treatment before Admission	Antibiotic Treatment at Time of Admission	Preoperative Aspiration	Microorganism Detected in Sample from Preoperative Aspiration	Microorganism Detected Intraoperatively	Surgical Treatment	Anti- Microbial Treatment	Duration Antibiotic Treatment (Days)	Implant Loosening	Number of Revisions	Reimplantation	Abduction (°)	Treatment Failure	Follow-Up (Months)
1	M	68	L	34.9	2	9	32.7	No	Fracture	Acute	Yes	Yes	No	n. a.	Staphylococcus epidermidis *	DAIR	Levofloxacin/ Clindamycin	70	No	2	n. a.	80	No	66
2	F	75	R	30.4	3	5	24.8	Yes	Osteoarthritis	Chronic	No	No	Yes	Not detected	Staphylococcus epidermidis *	TMS with spacer	Levofloxacin/ Clindamycin	45	Yes	3	No	70	Yes	82
3	F	60	R	34.6	3	5	31.7	No	Cuff-arthropathy	Acute	No	No	Yes	Not detected	Staphylococcus epidermidis	TMS without spacer	Levofloxacin/ Clindamycin	99	Yes	3	No	110	No	49
4	M	53	R	23	2	1	5	No	Fracture	Acute	No	No	No	n. a.	Staphylococcus epidermidis *	DAIR	Levofloxacin/ Clindamycin	28	No	2	n. a.	40	No	56
5	M	62	L	27.5	3	3	5	Yes	Fracture	Acute	No	No	No	n. a.	Staphylococcus epidermidis */ Enterococcus faecalis	TMS without spacer	Levofloxacin/ Clindamycin	65	Yes	4	Yes	60	No	54
6	F	68	R	39.1	2	3	11.3	No	Fracture	Acute	No	No	No	n. a.	Staphylococcus epidermidis */ Enterococcus faecalis/Proteus mirabilis	TMS with spacer	Vancomycin/ Rifampicin	214	Yes	12	No	50	Yes	54
7	F	93	R	20.7	3	8	331	No	Osteoarthritis	Chronic	No	No	Yes	Staphylococcus aureus	Staphylococcus aureus	TMS without spacer	Flucloxacillin/ Rifampicin	70	Yes	2	No	50	No	30
8	F	67	L	37.6	3	7	66.3	Yes	Fracture	Acute	No	No	No	n. a.	Staphylococcus aureus	DAIR	Cotrimoxazol/ Rifampicin	60	No	6	n. a.	40	No	109
9	M	50	L	20.2	2	2	311	No	Fracture	Acute	No	No	Yes	Staphylococcus aureus	Staphylococcus aureus	TMS with spacer	Ampicillin-Sulbactam/ Clarithromycin	52	No	5	Yes	60	No	114
10	M	59	L	27.5	3	7	110	No	Fracture	Acute	No	No	No	n. a.	Staphylococcus aureus	TMS with spacer	Flucloxacillin/Vancomycin	43	Yes	4	Yes	80	No	18
11	F	70	R	27.8	3	4	6.4	Yes	Osteoarthritis	Acute	No	No	No	n. a.	Staphylococcus aureus	DAIR	Levofloxacin/ Clindamycin	52	No	1	n. a.	60	No	18
12	F	48	R	21.2	2	1	5	No	Fracture	Chronic	No	No	Yes	Staphylococcus aureus *	Staphylococcus aureus *	TMS with spacer	Clindamycin/ Rifampicin	109	Yes	3	Yes	100	No	40
13	M	78	R	27	2	5	6.9	Yes	Fracture	Chronic	No	No	No	n. a.	Not detected	DAIR	Levofloxacin/ Clindamycin	38	No	3	n. a.	40	No	88
14	M	54	L	22	2	1	125	No	Osteoarthritis	Chronic	Yes	Yes	No	n. a.	Not detected	DAIR	Levofloxacin/ Rifampicin	49	No	1	n. a.	70	No	18
15	F	69	R	33.8	2	4	11.1	No	Cuff-arthropathy	Chronic	No	No	No	n. a.	Not detected	TMS with spacer	Levofloxacin/ Clindamycin	81	No	3	Yes	50	No	138
16	M	66	R	26.3	2	3	104	Yes	Osteoarthritis	Acute	Yes	Yes	No	n. a.	Not detected	TMS with spacer	Cefuroxim/ Rifampicin	101	Yes	2	Yes	60	No	18
17	F	79	R	22.4	2	5	67.2	Yes	Osteoarthritis	Acute	No	No	Yes	Escherichia coli	Escherichia coli	DAIR	Levofloxacin/ Clindamycin	47	No	3	n. a.	70	No	84
18	F	70	L	27.3	3	4	159	Yes	Osteoarthritis	Acute	No	No	No	n. a.	Escherichia coli/Citrobacter freundii	DAIR	Moxifloxacin/ Clindamycin	57	No	2	n. a.	30	No	40
19	F	67	L	25.4	2	3	5	No	Fracture	Chronic	No	No	No	n. a.	Cutibacterium acnes	TMS with spacer	Clindamycin	96	No	7	Yes	50	No	18

M male, F female, R right, L left, BMI body mass index, ASA American society of anesthesiologists, CCI Charlson Comorbidity Index, CRP C-reactive protein, DAIR debridement, antibiotics, irrigation, and retention of implant, TMS two/multiple stages, n. a. not applicable. * Methicillin-resistant.

## Data Availability

The data presented in this study are available on request from the corresponding author.
